# First Insights into Bioaccumulation Patterns in Different Tissues of the Greenland Shark *Somniosus microcephalus* from Kulusuk (Southeastern Greenland)

**DOI:** 10.3390/biology14070857

**Published:** 2025-07-15

**Authors:** Francesca Romana Reinero, Emilio Sperone, Samira Gallo, Donatella Barca, Francesco Luigi Leonetti, Gianni Giglio, Primo Micarelli

**Affiliations:** 1 Sharks Studies Centre-Scientific Institute, 58024 Massa Marittima, Italy; direzione@centrostudisquali.org; 2 Department of Biology, Ecology and Earth Sciences, University of Calabria, 87036 Rende, Italy; samira.gallo@unical.it (S.G.); donatella.barca@unical.it (D.B.); francescoluigi.leonetti@unical.it (F.L.L.); gianni.giglio@unical.it (G.G.); 3 Department of Physical Sciences, Earth and Environment, University of Siena, 53100 Siena, Italy

**Keywords:** elasmobranch, *Somniosus microcephalus*, environmental pollution, trace elements, Arctic Ocean

## Abstract

Marine environmental pollution has been rapidly increasing in Arctic waters, and the release and bioaccumulation of trace elements in Arctic marine species may pose significant risks to both ecosystem health and human well-being. As a top predator, the Greenland shark is an ideal sentinel species for ecotoxicological studies in this region. In this study, trace element analyses were conducted on various tissues from two Greenland sharks—a male and a female—collected in Kulusuk (southeastern Greenland). Eleven trace elements (Mn55, Co59, Cu63, Zn64, As75, Se82, Rb85, Mo98, Ag107, Cd112, and Pb208) were measured in different skin samples from both specimens and in the muscle and fat of the female using an Inductively Coupled Plasma Mass Spectrometer (ICP-MS). Principal Component Analyses (PCAs) revealed sex-related differences in skin bioaccumulation patterns, likely due to sampling of different skin regions. Notably, skin tissues from both sharks showed the highest concentration of trace elements, especially for As75 (9.39–41.13 ppm) and Zn64 (24.34–50.99 ppm) and with the exception of Ag107. These findings suggest that environmental exposure may play a more significant role than dietary intake in trace element accumulation in this area. This study represents the first investigation of trace element bioaccumulation in Greenland sharks from Kulusuk. While the results offer important preliminary insights into the species’ ecotoxicology, further research involving more specimens and tissues is needed to confirm these trends. These initial findings contribute to filling key data gaps and have implications for both environmental monitoring and public health within the Greenlandic community.

## Simple Summary

The Greenland shark, as a top predator in Arctic marine ecosystems, serves as an excellent sentinel species for ecotoxicological studies in the Arctic environment. This study aimed to investigate, for the first time, the bioaccumulation patterns of trace elements across different tissues in two Greenland sharks—a female and a male—collected from Kulusuk, southeastern Greenland. The results revealed both sex-related differences in trace element accumulation and a notably high concentration of pollutants in the skin, indicating that uptake likely occurs primarily through environmental exposure in the marine habitat. Considering that the Greenland shark is part of the local diet, these findings also have important implications for public health within the Greenlandic community.

## 1. Introduction

Over the past century, pollution and the influx of metals and metalloids into the marine environment have rapidly increased [1]. Among these pollutants, trace elements—particularly metals—are introduced through both natural and anthropogenic processes and pose a threat to marine animals and human health due to their toxicity and environmental persistence [2]. Bioaccumulation of trace elements in marine species can occur through direct contact or via the diet, and is influenced by various factors including tissue function, life stage, sex, reproductive status, and overall physiological exposure [3,4,5,6]. The World Health Organization (WHO) classifies trace elements into two categories: essential elements, which are vital for biological functions but can become toxic at high concentrations, and non-essential elements, which are often harmful even at low levels [7,8]. Some trace elements may biomagnify through food webs and accumulate in organisms throughout their lifespan without effective excretion [9]. Because of this, marine organisms are often used as bioindicators of contamination levels and ecosystem health [6,10].

Sharks are apex or meso-predators and play a crucial role in marine food webs; they are particularly susceptible to pollutant uptake and biomagnification, often representing sentinels for environmental contamination [2,5]. Their capacity to accumulate high levels of trace elements can negatively impact physiological processes and population dynamics [6,11].

The Greenland shark *Somniosus microcephalus* (Bloch and Schneider, 1801) is the largest fish species in the Arctic seas [12], and it has been recognized as the world’s longest-lived vertebrate [13,14]. Despite numerous studies addressing its biology [14,15,16,17] and ecology [18,19,20,21,22,23,24,25], ecotoxicological research on this species remains limited.

Due to its extreme longevity, slow growth, late maturity, low fecundity, and specific dietary habits [12,26], the Greenland shark is highly vulnerable to population-level impacts and pollutant bioaccumulation [13].

While data on contaminant bioaccumulation in Arctic shark species are scarce [27], the Greenland shark has emerged as a promising sentinel species for Arctic ecotoxicological studies [27,28,29]. Furthermore, the presence of contaminants in Arctic waters and their potential bioaccumulation in Arctic marine species could also be connected to the evolving climate change and the consequent increase in sea transports and oil, gas and mining activities in the fjords and on the shelf of the Arctic Ocean coasts [30].

Given the ecological role of the Greenland shark in Arctic marine ecosystems, this species bioaccumulates pollutants in target tissues both from the environment and the diet, and the bioaccumulation can be influenced by environmental and biological variables [12,27,30,31,32,33,34,35]. Various contaminants—including brominated and chlorinated flame retardants [31,32], fluorinated stain repellents [32], endocrine-disrupting compounds [30], organochlorines [33], persistent organic pollutants, and trace elements [12,27,32,34]—have been analyzed in Greenland shark tissues across Arctic regions. However, studies focused specifically on trace element bioaccumulation have been conducted only in Canadian [27,32,35], Icelandic [34], and northeastern Greenland populations [12], with no data available from southeastern Greenland.

For instance, the few studies concerning trace element bioaccumulation in the Greenland shark have largely been limited to hepatic concentrations of trace elements [27], mercury levels in selected tissues (e.g., cartilage, skin, muscle) [34,35], and trace element concentrations in other target tissues [12,32] in order to evaluate environmental and dietary exposure. Therefore, the bioaccumulation of non-essential biomagnifying and essential trace elements could provide information concerning diet, trophic level, and physiological requirements of an organism, while non-essential trace elements that have not been shown to biomagnify could provide information on geographical exposure variations [27]. Thus, having an overall view into concentrations and patterns of certain trace elements in the Greenland shark is a valid effort with potentially important implications both for sharks and human health.

Understanding the distribution and concentration of trace elements in Greenland sharks is therefore critical, with potential implications for both ecological health and human risk assessment. Given the concern about the toxicity of certain trace elements to Arctic wildlife and human populations [27], it is essential to monitor their accumulation in Arctic marine environments using sentinel species like the Greenland shark [30].

The aim of this study is to investigate, for the first time, the bioaccumulation patterns of trace elements in different tissues (skin, muscle, fat) of two Greenland sharks from Kulusuk (southeastern Greenland), evaluating bioaccumulation differences between sexes, tissues, and various skin regions from the same individual.

## 2. Materials and Methods

### 2.1. Sampling Area and Data Collection

**Figure 1:**
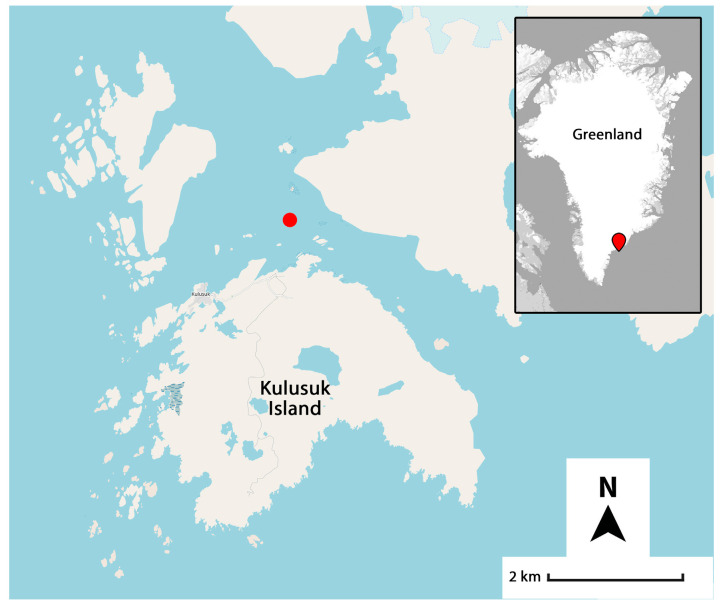
Kulusuk Island and the area (red dot) where Greenland sharks were observed and tissue samples collected.

Between 13 March and 20 March 2024, researchers of the Sharks Studies Centre-Scientific Institute of Massa Marittima (GR, Italy) conducted a pioneering expedition to Kulusuk, a small island covering 10.92 km^2^ in the southeastern part of Greenland (Figure 1). The main objective of the expedition was to observe the Greenland shark through diving-based field research.

Four dives were conducted off the coast of Kulusuk (65°38′4.848″ N, 37°11′20.436″ W, indicated by the red dot in Figure 1) where, on the frozen sea, a circular hole was cut through the one-meter-thick ice to give researchers the opportunity to dive and observe the Greenland shark in its natural environment. The Greenland shark was lured to the surface using baits lowered a few meters deep.

**Figure 2:**
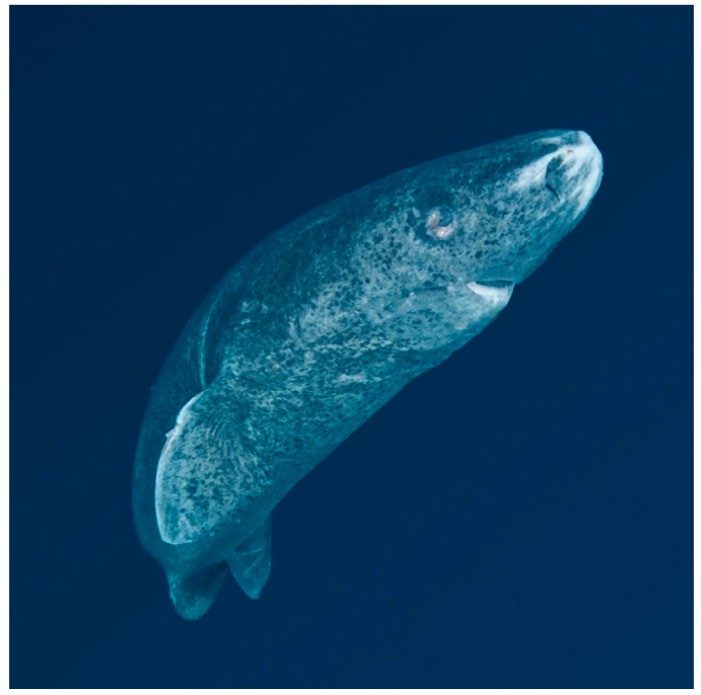
One of the two Greenland sharks, a female, observed at 3 m depth under the ice along the Kulusuk coast. Photo credits: Francesca Romana Reinero.

During the diving sessions, two different Greenland shark specimens were observed (Figure 2). Additionally, tissue samples were opportunistically collected from a recently caught female and a freshly caught male, both of which had been taken during subsistence harvesting by local hunters from the southeastern Greenlandic community of Kulusuk.

Due to the frozen condition of the recently caught female Greenland shark lying on ice, it was only possible to collect samples from the external epidermal area and the underlying muscle and fat in the caudal region. In contrast, only the external pelvic area of the freshly caught male was exposed above the ice, allowing for the collection of a single sample from pelvic fins.

Three skin samples from the caudal, dorsal and nostril areas and muscle and fat samples from the caudal area of the female were collected. One skin sample was taken from the left pelvic fin of the male. All samples were collected using a professional surgical scalpel.

An identification code was assigned to each sample (Sm01sc = skin taken from the caudal area of the female; Sm01sn = skin taken from the nostril area of the female; Sm01sd = skin taken from the dorsal area of the female; Sm01m = muscle taken from the caudal area of the female; Sm01f = fat taken from the caudal area of the female; Sm02spf = skin taken from the left pelvic fin of the male). Samples were immediately stored at −20 °C in Kulusuk and subsequently transported to the University of Calabria for ecotoxicological analyses.

**Figure 3:**
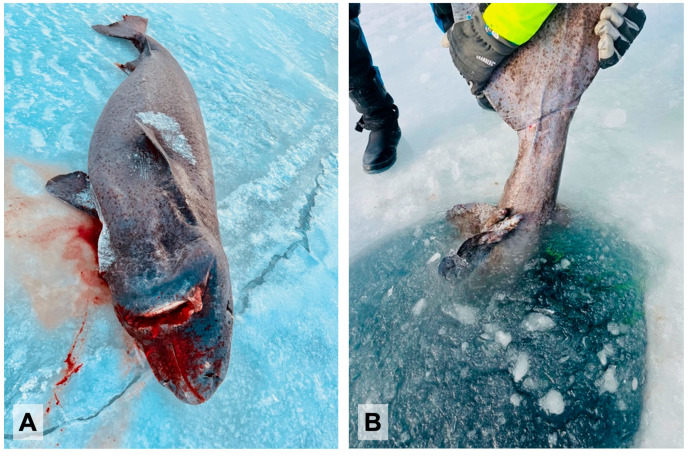
Female (**A**) and male (**B**) Greenland sharks from which samples were collected. Photo credits: Francesca Romana Reinero.

The sex of each specimen was determined through the presence (male) or absence (female) of claspers. The total length (TL) of the female, lying fully on the ice, was measured at 299 cm, indicating an immature individual [15]. Conversely, due to the male’s partial submersion—with only the caudal fin and pelvic area exposed—measuring its TL was not feasible (Figure 3).

### 2.2. Ecotoxicological and Statistical Analyses

Samples were first dehydrated under a laminar flow hood (Herasafe, model KS-12 class II) for 24 h and then weighed without pulverization in teflon digestion vessels on a high-precision analytical balance (sensitivity: 0.0001 g; Sartorius, model CP324S-0CE, Gottingen, Germany). Afterward, 12 mL of ultrapure nitric acid (HNO_3_; 64–69%) was added to each vessel and the acid digestion was catalyzed using an EMC microwave oven (MARS-6). The solution obtained was then placed on a plate at 200 °C in order to evaporate as many acid fumes as possible. Each concentrated solution was then transferred into glass flasks and diluted to a fixed volume (50 or 100 mL depending on the weight of the sample) with ultrapure water and stored in sterile containers at 4 °C. The same protocol (acid attack digestion by a microwave oven, evaporation on a hot plate, and dilution in ultrapure water) [6] was used to prepare the powder of the certified reference material (CRM; Tort-3; National Research Council Canada), which was used to evaluate the accuracy of the method during the analysis. Each sample was analyzed through the Elan DRC-e (Perkin Elmer/SCIEX) Inductively Coupled Plasma Mass Spectrometer (ICP-MS), which performs three internal replicates for each measurement; therefore, for every analyzed sample, the instrument generates three separate readings, which are then averaged to provide the final concentration value. This approach ensures both precision and reliability in the reported data.

In addition, during the analytical sequence, three determinations of TORT solution as quality control standards and three determinations of a blank solution (ultrapure water acidified by 2% of ultrapure nitric acid), interspersed at the beginning after 3 samples and at the end of the analytical run, were carried out to obtain the accuracy and detection limit of the elements. To evaluate the accuracies, the mean values of measurements carried out on the quality control standard (TORT) analyzed as unknown were compared with those certified. Accuracies, expressed as the relative differences from reference values, are listed in Table 1, as well as the instrument detection limit, which was evaluated for all analyzed elements on the blank solution by multiplying the standard deviation of the average blank by a factor of three.

In total, 11 trace elements were analyzed: manganese (Mn55), cobalt (Co59), copper (Cu63), zinc (Zn64), arsenic (As75), selenium (Se82), rubidium (Rb85), molybdenum (Mo98), silver (Ag107), cadmium (Cd112), and lead (Pb208). Of the analyzed elements, Mn55, Co59, Cu63, Zn64, Se82, As75, and Mo98 were considered essential or potentially essential for the Greenland shark based on its diet [27], while the others (Rb85, Ag107, Cd112, and Pb208) were considered non-essential. All the elements were expressed in ppm (µg/g).

Principal Component Analysis (PCA) was conducted to explore patterns in trace element bioaccumulation. Two separate PCAs were performed: one comparing element profiles in skin samples from the female (Sm01sc, Sm01sn, Sm01sd) and the male (Sm02spf), and another analyzing variability across different tissues from the female (skin: Sm01sc, Sm01sn, Sm01sd; muscle: Sm01m; fat: Sm01f). Data were scaled to unit variance prior to analysis. The biplots illustrate samples as points and elements as vectors, where the arrow direction indicates increasing concentration, the length represents the explained variance, and angles between vectors show associations. All analyses were performed using RStudio (Version 2024.12.1+563).

## 3. Results

The concentration values of 11 trace elements were obtained in all the tissues of both specimens (Table 1).

The concentration of each trace element in the target tissues, expressed in decreasing order and in percentages (%) in the districts, was as follows:

Mn55: Sm01sd (27.83%) > Sm01sc (25.8%) > Sm02spf (17.4%) > Sm01m (10.22%) > Sm01sn (9.67%) > Sm01f (9.08%).

Co59: Sm01sc (27.78%) > Sm01sd (25%) > Sm02spf (16.67%) > Sm01sn (13.89) > Sm01f (11.11%) > Sm01m (5.56%).

Cu63: Sm01sn (27.92%) > Sm01f (19.6%) > Sm01m (16.51%) > Sm02spf (14.23%) > Sm01sd (11.81%) > Sm01sc (9.93%).

Zn64: Sm01sd (22.62%) > Sm02spf (20.76%) > Sm01sn (18.45%) > Sm01sc (15.66%) > Sm01f (11.71%) > Sm01m (10.8%).

As75: Sm01sn (35.55%) > Sm02spf (15.38%) > Sm01sd (14.98%) > Sm01m (12.99%) > Sm01f (12.98%) > Sm01sc (8.12%).

Se82: Sm01sn (27.72%) > Sm02spf (22.8%) > Sm01sd (15.69%) > Sm01sc (12.13%) > Sm01f (11.61%) > Sm01m (10.04%).

Rb85: Sm01sn (29.38%) > Sm02spf (21.58%) > Sm01sd (18.16%) > Sm01sc (13.35%) > Sm01m (10.36%) > Sm01f (7.16%).

Mo98: Sm01sd (24.14%) > Sm01sn (20.69%) > Sm01sc (17.24%), Sm01f (17.24%) > Sm02spf (13.79%) > Sm01m (6.9%).

Ag107: Sm01m (45.76%)> Sm02spf (18.64%) > Sm01sn (13.56%)> Sm01sc (8.47%), Sm01sd (8.47%) > Sm01f (5.08%).

Cd112: Sm01sn (28.74%) > Sm01sd (27.59%) > Sm01sc (14.94%) > Sm01f (13.79%) > Sm01m (11.49%) > Sm02spf (3.45%).

Pb208: Sm01sc (38.85%) > Sm01sn (14.39%), Sm02spf (14.39%) > Sm01sd (14.03%), Sm01f (14.03%) > Sm01m (4.32%).

Trace elements were observed in higher concentrations in female skin samples (Sm01sn, Sm01sc, Sm01sd), except for Ag107, which was more abundant in the muscle (Sm01m). For instance, elements like Cu63, As75, Se82, Rb85, and Cd112 accumulated more in the female skin from the nostril area (Sm01sn), Mn55, Mo98, and Zn64 in the female skin from the dorsal area (Sm01sd), and Co59 and Pb208 in the female skin from the caudal area (Sm01sc).

In muscle (Sm01m) and fat (Sm01f), trace elements generally accumulated less compared to skin samples, where concentrations of Zn64 and As75 showed the highest values.

**Figure 4:**
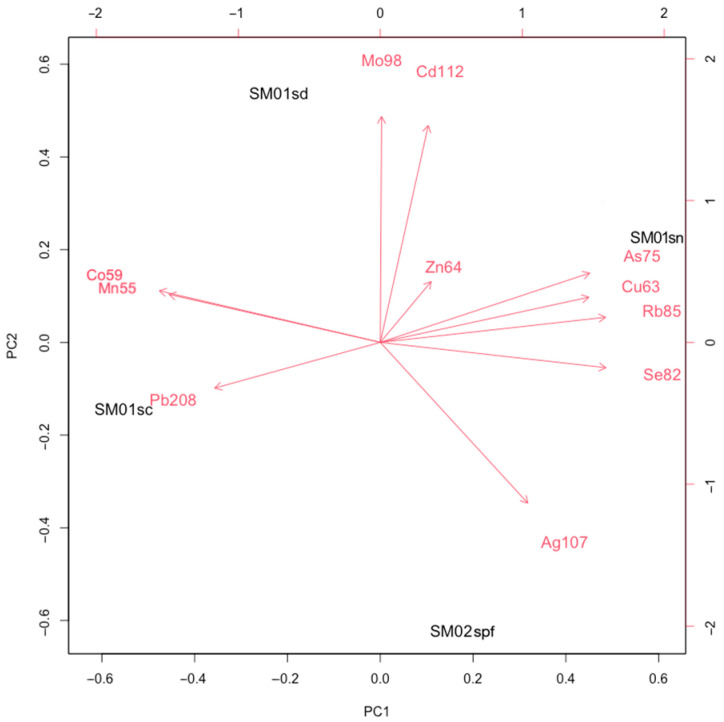
Biplot from the Principal Component Analysis (PCA1) showing the patterns of trace element bioaccumulation in skin samples from female (Sm01sc, Sm01sn, Sm01sd) and male (Sm02spf).

In the first PCA test, two principal components (PC1 and PC2) explain a high percentage of the total variance (84.55%) (Table 2), and female and male skin samples show differences in bioaccumulation patterns (Figure 4).

As concerns the female samples, Pb208 tends to accumulate in the skin from the caudal area (Sm01sc) and Mo98 and Cd112 in the skin from the dorsal area (Sm01sd), while the skin from the nostril area (Sm01sn) and the skin from the dorsal area (Sm01sd) show different bioaccumulation patterns than the skin from the pelvic fin of the male (Sm02spf). Furthermore, there is a positive association between Zn64, As75, Cu63, Rb85 and Se82 in all the analyzed skin districts, and Pb208 concentrations tend to vary in the opposite way to Zn64, As75, Cu63, Rb85, and Se82, and the same occurs between Ag107 and Co59 and Mn55.

**Figure 5:**
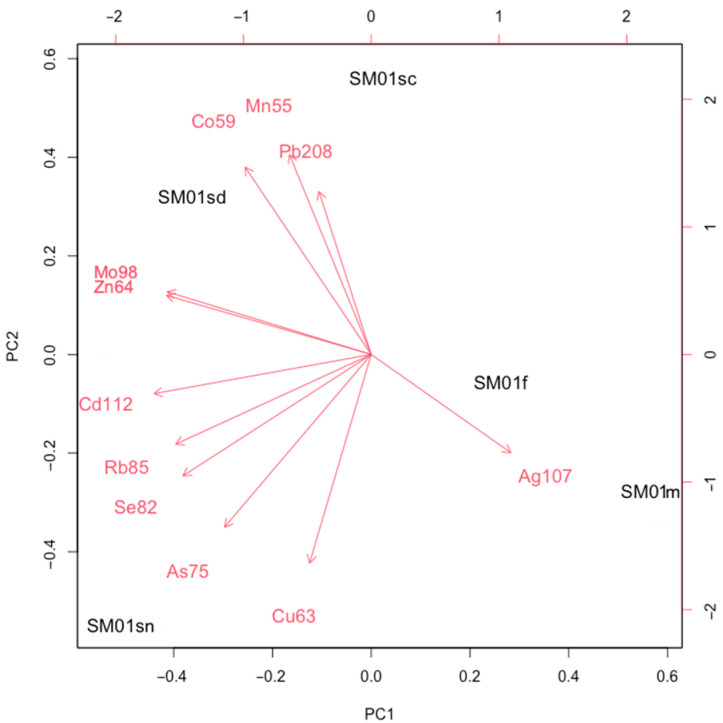
Biplot from the Principal Component Analysis (PCA2) illustrating the variability in trace element bioaccumulation across different tissues (Sm01sc, Sm01sn, Sm01sd, Sm01m, Sm01f) from the female.

In the second PCA test, two principal components (PC1 and PC2) explain a high percentage of the total variance (85.46%) (Table 3), and female tissues show differences in bioaccumulation patterns (Figure 5).

In particular, Mn55, Co59, and Pb208 tend to accumulate in the skin from the caudal area (Sm01sc), while Mo98 and Zn64 accumulate in the skin from the dorsal area (Sm01sd). Furthermore, the skin from the nostril area (Sm01sn) shows a possible association with higher concentrations of Cd112, Rb85, Se82, As75, and Cu63, while fat (Sm01f) and muscle (Sm01m) are strictly associated with Ag107 bioaccumulation. Concentrations of Ag107 tend to vary in the opposite way to Mo98 and Zn64.

## 4. Discussion

This study represents the first investigation into trace element bioaccumulation patterns in Greenland shark tissues from the southeastern Greenland region of Kulusuk.

Due to the unavailability of TL data for the male specimen, comparisons of bioaccumulation patterns in relation to shark size were not feasible. McMeans et al. [27] in Canada found no correlation between trace element bioaccumulation in Greenland sharks and their size, likely due to the limited size range of the studied individuals or the lack of a direct increase in trophic level with growth. In contrast, Nielsen et al. [23] reported ontogenetic dietary shifts in Greenland sharks, with smaller individuals feeding on lower-trophic-level prey such as squids, and larger sharks consuming higher-trophic-level prey like seals and benthic fishes. These findings underscore the need for further studies in Kulusuk that assess bioaccumulation patterns across a broader range of specimens and life stages to better understand the ecological and toxicological implications.

The difference in trace element concentrations between skin samples of the male (Sm02spf) and those from the female—especially the nostril (Sm01sn) and dorsal (Sm01sd) regions—may be attributed to either variation in skin sampling locations or to sex-related biological and ecological factors. Additional comparative data from multiple individuals, sampled at consistent anatomical locations, are necessary to investigate sex-based bioaccumulation trends. Mc Means et al. [27] analyzed 24 Greenland shark livers in Canada, and found sex-specific differences in only 1 of 24 analyzed trace elements (Rb85), with higher levels detected in females. As Rb85 is non-essential and known to biomagnify in Arctic marine food webs [36], this sex-specific accumulation may be attributed to different exposure levels or physiological regulation capabilities between sexes [37].

Corsolini et al. [12] remain the only researchers to date to report concentrations of Cd112, Pb208, and Se82 in the skin and muscle of this species from the northeastern part of Greenland. They observed higher levels of these elements in skin compared to muscle, a trend confirmed by the present study. This supports the hypothesis that the skin may play a more active role in trace element uptake, which has implications for local dietary safety, as Kulusuk residents typically consume only the muscle tissue.

To date, no studies have systematically evaluated trace element homogeneity across shark skin at a whole-body scale. Bryan et al. [38] demonstrated longitudinal (dorsal–ventral), but not lateral (anterior–posterior), variability in trace element concentrations in the bottlenose dolphin *Tursiops truncatus* (Montagu, 1821). This suggests that skin from different body regions, such as those sampled from the female Greenland shark in Kulusuk, could exhibit spatial heterogeneity. More specimens must be analyzed to determine whether this is a generalizable pattern in sharks.

Among the analyzed elements, Zn64 and As75 exhibited the highest concentrations across all tissues, consistent with the results from McMeans et al. [27] and Muir et al. [32] on Greenland sharks from Canada. However, levels in Kulusuk specimens were notably higher. This discrepancy may reflect differences in the tissue types sampled (only muscle overlapped across the studies), dietary composition, local anthropogenic activities, or oceanographic factors such as salinity, sediment load, and currents.

The elevated concentration of Zn64 in the female dorsal skin (Sm01sd) suggests environmental absorption, as Zn64 is abundant in the deep waters of the northeast Atlantic [39], and aqueous uptake of Zn64 is more important than dietary intake for elasmobranchs, due to the high affinity of their skin to this element [40,41]. However, the relatively high levels found in muscle (Sm01m) and fat (Sm01f) also indicate dietary intake. For instance, the Greenland shark, in Greenland waters, preys mainly on Atlantic cod, harp seal, skates and wolffish [22], which are known Zn64 sources [42,43,44]. Indeed, this essential element is commonly bioaccumulated in elasmobranchs through the diet, and it has a fundamental role in a wide range of physiological processes, showing an increase during the growth of fish [27] and the diet shift of sharks, probably due to the greater predation of larger prey [45]. Although this element has been suggested to biomagnify [37], it seems to be efficiently regulated by fish, and the variance in Zn64 in the food web could be linked to different physiological requirements, with higher Zn64 requirements in higher-trophic-level vertebrates, like the Greenland shark [27].

As75 was found in high concentrations in the female nostril skin (Sm01sn), as well as in muscle and fat, suggesting both environmental and dietary exposure. As75 is common in the Arctic food web, particularly in seals, fish and birds [36,46,47], prey of the Greenland shark. In previous studies, the analysis of As75 in the liver and muscle of different demersal and pelagic sharks revealed a positive relationship between different stages of the biological cycle and accumulation of As75 in the muscle [48], while this relationship was negative in the liver [49]. Furthermore, high concentrations of As75 were detected in the muscle tissue of the Greenland shark from Quebec, Canada [32], as well as in the same tissue of the female specimen analyzed in this study. However, As75 does not appear to biomagnify within the Arctic food web [36]. This suggests that the element, which may be potentially essential, undergoes metabolization and detoxification in the liver of sharks [47,50], which could explain the lower levels observed in the livers of Greenland sharks from Cumberland Sound [27] and Quebec [32]. Regarding As75 accumulation in skin samples, mining activities in Greenland have caused significant metal pollution, including along the country’s eastern coast [48,51]. Elevated concentrations of As75 have been reported in mine waste and have also been found to accumulate in green sea urchins located far from the mining sites [49,52]. Therefore, the high concentrations of As75 in Greenland waters may contribute to substantial environmental bioaccumulation of this element in Greenland sharks, although dietary intake likely also plays a relevant role.

The Greenland shark commonly bioaccumulates Rb85, although its physiological role is still not fully clear [27]. Higher levels of Rb85 found in the skin (Sm01sn) rather than in muscle (Sm01m) and fat (Sm01f) and in other tissues of this species from Canada [27,32] may show high bioaccumulation in dermal denticles and an environmental exposure in Kulusuk. Unfortunately, data on the marine environmental concentration of Rb85 in this area are not available. This non-essential element is known to increase with increasing trophic level, and it is less efficiently regulated than essential elements [36], suggesting its variances are indicative of environmental exposure differences between the areas.

Mn55 concentrations in skin (Sm01sc, Sm01sd) and muscle (Sm01m) exceeded those reported by Muir et al. [32] and McMeans et al. [27]. While Mn55 is an essential micronutrient typically acquired through the diet [53], the elevated levels in this study may reflect poor metabolic regulation or high environmental availability.

Despite the fact that Cu63 tends to accumulate in the skin (Sm01sn), its concentration in the muscle (Sm01m) was higher than in the same tissue of the Greenland shark from Quebec [32], probably due to the sharks’ different diets in the areas, and it was more accumulated in the liver of sharks from Cumberland Sound [27]. Cu63 is common in Greenland shark prey [43] and accumulates in the liver, where it is detoxified by metallothioneins along with Zn64 and Cd112 [54].

Se82 followed known patterns, with the highest concentration in skin (Sm01sn) and lower levels in muscle and fat, consistent with Corsolini et al. [12], McMeans et al. [27], and Muir et al. [32]. Se82 plays a protective role against mercury toxicity [12], and its relatively low muscle concentration may indicate either low mercury levels or higher Se82 storage in organs like the pancreas or spleen [12].

The concentration of Cd112 in the skin (Sm01sn and Sm01sd) could indicate its affinity with dermal denticles [41], and it was found in high concentration in the deep waters of the northeast Atlantic [39,55]. In the skin, Cd112 values were consistent with the results of Corsolini et al. [12] and Muir et al. [32]. However, higher concentrations of Cd112 were detected in other organs (i.e., pancreas, liver, spleen, brain) rather than in the skin of Greenland sharks from other areas, indicating this element could be mainly related to their diet, particularly at high latitudes [12,43,56]. Indeed, Cd112 has been observed to exhibit trophic transfer in some species and to biomagnify [54], and large concentrations of this non-essential trace element are commonly found in the liver of sharks, with levels positively increasing as the animal increases in size. Marcovecchio et al. [54] also suggested that high concentrations of Zn64 are linked to the presence of Cd112, since Zn64 in the liver would exert a protective effect against Cd112 toxicity, detoxifying the organism.

Also, levels of Pb208 in the skin (Sm01sc) were consistent with the results obtained by Corsolini et al. [12] since this element is highly present in Greenland waters [55]. It may be mainly absorbed by the dermal denticles, which have a rough surface favorable to the attachment of particulates or sediment [12]. However, despite the fact that concentrations of Pb208 in the muscle (Sm01m) are low and show similar values to those observed by Muir et al. [32] and Corsolini et al. [12], this non-essential element could be accumulated by prey of the Greenland shark [43]. However, concentrations in other target tissues of Greenland sharks from Greenland [12] and Canada [32] are quite below the values observed in the skin.

Given the human health concern about the toxicity of some non-essential trace elements (Cd112, Pb208) in food consumption, the observed results in the muscle (Sm01m), originally expressed on a dry weight (d.w.) basis, were converted to wet weight (w.w.) concentrations using the formula provided by Gaion et al. [57]. The water loss percentage used for this conversion was 70.45%, as reported for shark muscle by Gallo et al. [6]. Accordingly, the concentrations of Cd112 and Pb208, the only two elements along with mercury (not considered in this study) regulated in fish muscle under European legislation, were found to be 0.03 ppm w.w. (vs. 0.1 ppm d.w.) for Cd112 and 0.04 ppm w.w. (vs. 0.12 ppm d.w.) for Pb208. When compared to the maximum limits currently in force under Commission Regulation (EU) 2023/915 [58], both elements were well below the permitted thresholds for food consumption. Thus, the Kulusuk community, which feeds on the Greenland shark meat, does not seem to be intoxicated by these elements, although more samples from more specimens are required to address this topic.

Levels of Ag107 in muscle (Sm01m) are higher than those in the muscle of the Greenland shark from Quebec [32]. This could be due to regional differences in exposure [27] since this non-essential element has not been observed to biomagnify in food webs [36].

Both Co59 and Mo98 showed general low values in the skin, and concentrations in the muscle (Sm01m) were slightly higher than those found in the shark from Quebec [32] and similar to those in the liver of sharks from Cumberland Sound [27]. Given the lower concentrations at which both Co59 and Mo98 were detected in other tissues [32], this evidence could highlight the general low presence of these essential elements in Greenland shark prey and in the Kulusuk marine environment.

In conclusion, the positive association between Zn64, As75, Cu63, Rb85 and Se82 in all the analyzed skin districts implies that some trace elements could be introduced into the study area through similar geochemical pathways [1]. Further direct experimental studies assessing both the biological and environmental factors that drive metal associations and their variation in the opposite way (i.e., Pb208 and Zn64, As75, Cu63, Rb85, Se82; Ag107 and Co59, Mn55, Mo98, Zn64) are required.

## 5. Conclusions

This study presents the first data on bioaccumulation patterns in the Greenland shark from Kulusuk, although the sample size was very limited, posing restrictions to the addressed findings. Thus, further studies involving a larger number of individuals and tissue samples are recommended to confirm the preliminary trends observed. Considering the limits posed by this study, the skin was revealed to be the tissue where the highest concentration of trace elements was detected with respect to muscle and fat, at least for the female. This preliminary finding could suggest that environmental pollution represents a source for trace element bioaccumulation in this area, although more different tissues from several animals and more investigations on the presence of trace elements in the waters of southeastern Greenland are required to further investigate this aspect. Nonetheless, muscle and fat were revealed to be important tissues for trace element bioaccumulation, not excluding the intake of pollutants through the diet also. This addresses health concerns among Kulusuk people who feed on the meat of this shark, although the concentrations of potentially toxic non-essential elements did not exceed legally permitted thresholds for human consumption. Sex differences found in skin bioaccumulation patterns require further studies in order to demonstrate that males accumulate trace elements differently than females, at least for different skin samples. Considering these findings, this study highlights the vulnerability of the Greenland shark to the bioaccumulation of trace elements in Kulusuk waters. Given the important ecological role of this species in the Arctic marine ecosystem, a deeper knowledge of the regulation, assimilation, and trophic transfer of trace elements is crucial, in addition to defining possible synergistic effects among different pollutants in ecotoxicological evaluations of this species in a global change scenario. This could be useful in addressing concerns not only about the ecotoxicology and physiology of this species, but also the human health of the Greenland community that feeds on the Greenland shark. Furthermore, conservation strategies aimed at fighting against global warming, reducing industrial production, which is a source of pollutants in the Arctic Ocean, and initiating research programs to better understand the distribution areas of this species in Kulusuk in order to avoid its decline by climate change and have an overall view on fishing activity for food consumption are recommended.

## Figures and Tables

**Table 1:** Trace element concentrations (ppm) in target tissues. The detection limit (DL) and the mean accuracy (mA) are reported for each trace element. It was not possible to calculate the mA of Rb85 and Ag107 since the certified values of these elements are not available.

Trace Element	DL	mA	Female Caudal Skin (Sm01sc)	Female Nostril Skin (Sm01sn)	Female Dorsal Skin (Sm01sd)	Female Muscle (Sm01m)	Female Fat (Sm01f)	Male Pelvic Fin Skin Sm02spf
**Mn55**	0.02	−4.86	6.11	2.29	6.59	2.42	2.15	4.12
**Co59**	0.001	−11.58	0.10	0.05	0.09	0.02	0.04	0.06
**Cu63**	0.34	−1.53	0.74	2.08	0.88	1.23	1.46	1.06
**Zn64**	0.50	−7.67	35.29	41.60	50.99	24.34	26.40	46.80
**As75**	0.06	1.53	9.39	41.13	17.33	15.03	15.01	17.79
**Se82**	0.25	2.71	1.16	2.65	1.50	0.96	1.11	2.18
**Rb85**	0.40	-	1.25	2.75	1.70	0.97	0.67	2.02
**Mo98**	0.01	−8.05	0.05	0.06	0.07	0.02	0.05	0.04
**Ag107**	0.01	-	0.05	0.08	0.05	0.27	0.03	0.11
**Cd112**	0.02	8.72	0.13	0.25	0.24	0.10	0.12	0.03
**Pb208**	0.001	1.68	1.08	0.40	0.39	0.12	0.39	0.40

**Table 2:** Importance of principal components derived from the Principal Component Analysis (PCA1), performed on trace element concentrations in skin samples, comparing female (Sm01sc, Sm01sn, Sm01sd) and male (Sm02spf). The standard deviation, proportion of variance, and cumulative proportion for each component are reported.

Component	Standard Deviation	Proportion of Variance	Cumulative Proportion
PC1	2.5547	0.5933	0.5933
PC2	1.6654	0.2521	0.8455
PC3	1.3037	0.1545	1.0000
PC4	3.272 × 10^−16^	0.0000	1.0000

**Table 3:** Importance of principal components derived from the Principal Component Analysis (PCA2), performed on trace element concentrations across different tissues (Sm01sc, Sm01sn, Sm01sd, Sm01m, Sm01f) from the female. The standard deviation, proportion of variance, and cumulative proportion for each component are reported.

Component	Standard Deviation	Proportion of Variance	Cumulative Proportion
PC1	2.2916	0.4774	0.4774
PC2	2.0370	0.3772	0.8546
PC3	0.98041	0.08738	0.94200
PC4	0.7987	0.0580	1.0000
PC5	3.345 × 10^−16^	0.0000	1.0000

## Data Availability

The raw data supporting the conclusions of this article will be made available by the authors on request.

## Abbreviations

The following abbreviation is used in this manuscript:

WHO	World Health Organization
